# De Novo Assembly and Species-Specific Marker Development as a Useful Tool for the Identification of *Scutellaria* L. Species

**DOI:** 10.3390/cimb43030152

**Published:** 2021-12-01

**Authors:** Hakjoon Choi, Wan Seok Kang, Jin Seok Kim, Chang-Su Na, Sunoh Kim

**Affiliations:** 1 Central R&D Center, B&Tech Co., Ltd., Gwangju 61239, Korea; ohchj12@naver.com (H.C.); kws2602@hanmail.net (W.S.K.); keki2000@naver.com (J.S.K.); 2 College of Korean Medicine, Dongshin University, Naju-si 58245, Korea; nakugi@hanmail.net

**Keywords:** *Scutellaria* L., de novo, sequencing, DNA barcode, specific primer

## Abstract

*Scutellaria* L. (family *Lamiaceae*) includes approximately 470 species found in most parts of the world and is commonly known as skullcaps. *Scutellaria* L. is a medicinal herb used as a folk remedy in Korea and East Asia, but it is difficult to identify and classify various subspecies by morphological methods. Since *Scutellaria* L. has not been studied genetically, to expand the knowledge of species in the genus *Scutellaria* L., de novo whole-genome assembly was performed in *Scutellaria indica* var. *tsusimensis* (H. Hara) Ohwi using the Illumina sequencing platform. We aimed to develop a molecular method that could be used to classify *S.* *indica* var. *tsusimensis* (H. Hara) Ohwi, *S. indica* L. and three other *Scutellaria* L. species. The assembly results for *S.* *indica* var. *tsusimensis* (H. Hara) Ohwi revealed a genome size of 318,741,328 bp and a scaffold N50 of 78,430. The assembly contained 92.08% of the conserved BUSCO core gene set and was estimated to cover 94.65% of the genome. The obtained genes were compared with previously registered *Scutellaria* nucleotide sequences and similar regions using the NCBI BLAST service, and a total of 279 similar nucleotide sequences were detected. By selecting the 279 similar nucleotide sequences and nine chloroplast DNA barcode genes, primers were prepared so that the size of the PCR product was 100 to 1000 bp. As a result, a species-specific primer set capable of distinguishing five species of *Scutellaria* L. was developed.

## 1. Introduction

*Scutellaria* L. is a genus of annual or perennial herbaceous plants belonging to the family *Lamiaceae*. Members of this genus live in temperate regions and approximately 470 species are known [1,2,3], with some used as folk remedies in Korea and East Asia. To date, it has been confirmed that extracts of the genus *Scutellaria* have antitumor, hepatoprotective, antioxidant, anti-inflammatory, anticonvulsant, antibacterial and antiviral effects [4]. In Korea, 17 species of the genus *Scutellaria* are known to grow wild and several subspecies have been reported in addition to these species [5]. However, subspecies of *Scutellaria* L. may be confused because of their morphological similarities. For this reason, certain *Scutellaria* L. species used in pharmaceuticals and foods are very commonly used interchangeably. Therefore, it is necessary to classify and identify these taxa molecularly rather than morphologically. *Scutellaria baicalensis* Georgi is a representative species of the genus *Scutellaria* and has efficacy in diarrhea, dysentery, hypertension, hemorrhaging, insomnia, inflammation and respiratory infections. *Scutellaria barbata* D. Don is also known as “Ban Zhi Lian” in China and “Banjiryun” in Korea, has been used for inflammations, hemoptysis, hematuria, stomach pain and mosquito bites. *Scutellaria indica* L. was employed for analgesia, detoxification, promoting blood circulation effects in China, Korea and India. The differences in efficacy traditionally used for each of these species actually led to differences in the constituent compounds. In addition, *Scutellaria indica* L., *Scutellaria indica* var. *tsusimensis* (H. Hara) Ohwi and *Scutellaria pekinensis* var. *transtra* (Makino) H. Hara is commonly referred to as “skullcaps”. These are subspecies with very similar phenotypes and are easily mixed and misused. For this reason, accurate identification and classification of each species is required not only in academia but also in industrial fields such as drug development using natural products [4,6]. However, genetic research on the genus *Scutellaria* is not active and only a small number of genes are registered in the NCBI GenBank database.

Molecular markers have been widely applied as useful tools to assess genetic variation or to classify species and have improved the genetic analysis of plants [7]. These molecular markers include random amplified polymorphic DNA (RAPD), ribosomal DNA (rDNA), simple sequence repeat (ISSR), sequence characterization amplification region (SCAR) and simple sequence repeat (SSR) markers [8,9,10,11,12,13,14]. In particular, SSRs are used as genetic markers for studies such as parentage analysis, fingerprinting, genetic structural analysis and genetic mapping due to their reproducible polymorphic features [15,16,17].

Next-generation sequencing (NGS) technology is advancing rapidly to create sequencing libraries and build effective solutions for data analysis [15]. Deep sequencing and de novo assembly using RNA sequencing technology has become an alternative to genomic sequencing for protein discovery and phylogenetic and evolutionary analysis in organisms [18]. Furthermore, it has accelerated the development of genome-wide SSRs or microsatellites that can be used more quickly and efficiently, even in non-model plants with limited background genetic information. For this reason, NGS is performed for the development of genetic markers. Recently, the successful generation of transcriptome data through NGS for the development of SSR markers in non-model plants lacking a reference genome has been reported successfully [7,14,19].

In this study, we performed de novo whole-genome assembly of *Scutellaria indica* var. *tsusimensis* (H. Hara) Ohwi. We aimed to develop an accurate set of markers for identification through genetic analysis of *S. indica* L. and *S. indica* var. *tsusimensis*, which are difficult to distinguish visually.

## 2. Material and Methods

### 2.1. Sampling and Genomic DNA Extraction

Samples of five *Scutellaria* L. species with different leaf shapes were collected from Gangjin County (Jeollanamdo, Korea). Total genomic DNA was extracted from five dried *Scutellaria* L. leaves (100 mg) using a Plant DNeasy Extraction Kit (Qiagen, Hilden, Germany). Sample 3 additionally extracted RNA for WGS, and RNA extraction was performed using a universal RNA extraction kit (Bioneer, Daejeon, Korea) following the manufacturer’s protocol. Only Sample 3 was performed WGS, and the DNA of the other four samples was used only for the identification of species-specific primers.

### 2.2. Polymerase Chain Reaction (PCR) Amplification

The reaction for PCR amplification using primers included 0.5 μL of Taq polymerase (nTaq Mg^2+^, Enzynomics, Daejeon, Korea), 2 μL of 10× nTaq buffer, 2 μL of dNTP mixture (2.5 mM), 1 μL of forward primer (10 pmole), 1 μL of reverse primer (10 pmole) and 2 μL of quantified template DNA (5 ng/μL) mixed together, and purified water was added to reach a final reaction volume of 20 μL. PCR conditions included predenaturation at 95 °C for 10 min, denaturation at 95 °C for 30 s, annealing at 42–55 °C for 30 s, amplification at 72 °C for 30 s and denaturation. After repeating the process up to amplification 30 times, a final extension step was performed at 72 °C for 10 min to obtain a DNA amplification product. The DNA amplification product was dripped on a 2% agarose gel and electrophoresed for 30 min, and when species specificity was confirmed, it was used for species differentiation.

### 2.3. Sequel Library Construction

A sample of high-quality and high-molecular-weight DNA is required to prepare size-selected (approximately 20 kb) SMRTbell templates. We used PicoGreen to measure the concentration of genomic DNA. All samples passed the QC screening criteria. For PacBio Sequel sequencing, 8 μg of input genomic DNA was used for 20 kb library preparation. For gDNA with a size range less than 17 kb, we used a Bioanalyzer 2100 (Agilent Technologies, Palo Alto, CA, USA) to determine the actual size distribution. The library insert sizes were in the optimal size range, and we sheared gDNA with a g-TUBE (Covaris Inc., Woburn, MA, USA) and purified it using AMPurePB magnetic beads (Beckman Coulter Inc., Brea, CA, USA) if the apparent size was greater than 40 kb. A 10 μL library was prepared using the PacBio SMRTbell Express Template Prep Kit 2.0 (Pacific Biosciences, Menlo Park, CA, USA). SMRTbell templates were annealed using Sequel Binding and Internal Ctrl Kit 3.0 (Pacific Biosciences). The Sequel Sequencing Kit 3.0 and SMRT Cells 1M v3 Tray were used for sequencing. Movies (600 min) of each SMRT Cell (Pacific Biosciences) were captured using the PacBio Sequel (Pacific Biosciences) sequencing platform by Macrogen Inc. (Seoul, Korea).

For the analysis, Illumina short read sequences were generated by the Illumina HiSeq XTen platform and PacBio long read sequences were generated by the PacBio Sequel system. Illumina short reads were assembled into contigs using Platanus-allee 2.2.2 [20] with a 51 initial k-mer size and 0.8 maximum k-mer factor. The contigs were subjected to scaffolding and gap filling using PacBio long read sequences by PBJelly 15.8.24 [21]. To assess assembly quality, BUSCO [22] (based on evolutionarily informed expectations of the gene content of near-universal single-copy orthologs) analysis was performed using BUSCO 3.0 with eukaryote odb version 9. Additionally, Illumina short reads were aligned against the gap-filled scaffolds to calculate the mapping ratio, mapping coverage and insert size for assessment. MAKER 3.01.03 [23] was used to predict genes from the gap-filled scaffolds based on the SNAP model. For additional annotation, the consensus sequences were searched against the GenBank nonredundant (NR), UniProt, GO, InterPro, Pfam, CD, TIGRFAM and EggNOG 4.5 databases [24] using blast [25] v2.6.0+.

### 2.4. Preparation of Species-Specific Primers Using Barcode DNA

The chloroplast DNA (cpDNA) barcodes of five species of *Scutellaria* registered in the NCBI GenBank database were analyzed. Specific primers were designed by aligning each nucleotide sequence with CLC Sequence Viewer to search for nucleotide sequences (single nucleotide polymorphisms (SNPs), deletions, insertions) that appeared only in the relevant species. To increase specificity when preparing a specific primer, a SNP was placed at the 3′-end, and the third nucleotide sequence was mismatched to the 5′-side therefrom to prepare a specific primer. In addition, the nucleotide sequence obtained through WGS de novo assembly was matched with the previously registered nucleotide sequence of the genus *Scutellaria* using the NCBI BLAST service. With the matching result, the primer was designed so that the PCR amplification product was 100 bp to 1000 bp using Primer-BLAST.

## 3. Results

### 3.1. Leaf Profiles of Five Species of Scutellaria L.

**Figure 1:**
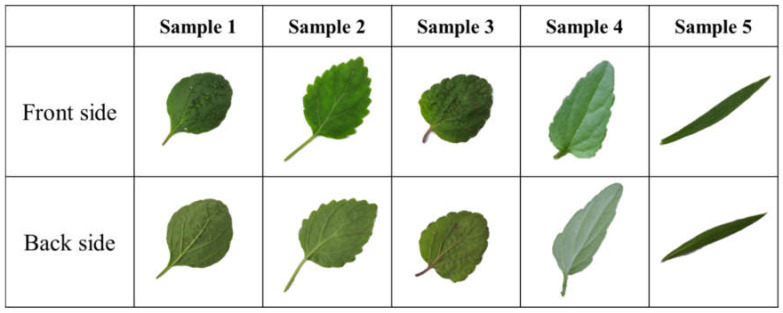
Leaf profiles of five species of *Scutellaria* L.

The leaf shape of five different species of *Scutellaria* L. is shown in Figure 1. Sample 1 has medium-sized leaves of 2–3.5 cm. The leaves are thin, there is almost no depression of the upper leaf veins, and the leaves are heart-shaped, almost round. Sample 2 has large leaves of 1.3–3.8 cm. The pulses on the upper side are not clear but the pulses on the lower side are protruding. The leaf is cordate, the leaf apex and base are acute, and the edge of the leaf has approximately 10 pairs of sharp serrated teeth. Sample 3 is very similar to Sample 1 but the veins are distinct, and the leaf shape is closer to the heart shape than that of Sample 1. The petiole of Sample 4 is narrow triangular-ovate to slightly tentacle-shaped, 1.2–2.5 cm long and 0.6–1.5 cm wide. It is apically acute, basal broadly wedge-shaped to sharp, with margins dull and shallow, 3–4 toothed, matte, and rarely with down along the veins. Sample 5 leaves are lanceolate with a length of 2.7–5.2 cm and a width of 0.6–1.1 cm, the leaf apex is acute, and the leaf base is rounded. In particular, it is very difficult to distinguish the species because the leaf size, shape and stem characteristics of Sample 1 and Sample 3 are very similar. Combining their characteristics, Sample 1 and Sample 3 are *S. indica* var. *tsusimensis* (H. Hara) Ohwi or *S. indica* L. Sample 2 has the characteristics of *S. pekinensis* var. *transitra* (Makino) H. Hara. Sample 4 has the characteristics of *S. barbata* D. Don. Sample 5 has the characteristics of *S. baicalensis* Georgi. However, their species could not be determined by the shape of the leaves. Therefore, a method was developed to confirm the species through whole-genome sequencing (WGS) analysis of Sample 3, and to use it to confirm the species by using the genetic characteristics of other species.

### 3.2. RNA-Seq-Based De Novo Assembly of Sample 3

**Figure 2:**
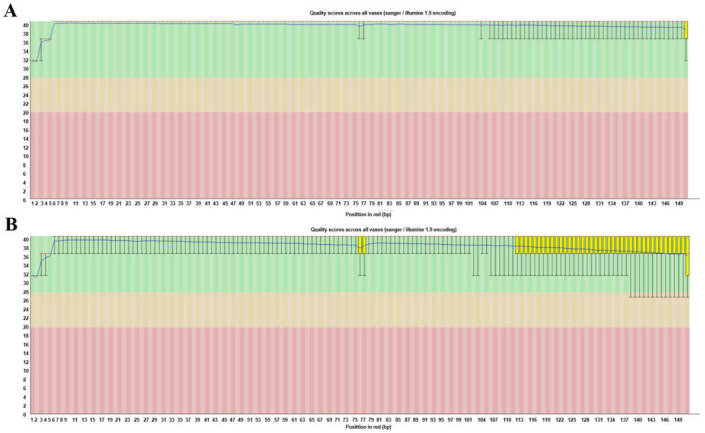
Base quality of DNA at each cycle after filtering. (**A**) Read 1 at each cycle after filtering. (**B**) Read 2 at each cycle after filtering. Yellow box: Interquartile range (25–775%) of Phred scores at each cycle. Red line: Median Phred score at each cycle. Blue line: Average Phred score at each cycle. Green background: Good quality. Orange background: Acceptable quality. Red background: Poor quality.

The total number of bases and reads, and GC (%), Q20 (%) and Q30 (%) values were calculated for the DNA sample. From the DNA, 973,624,734 reads were produced, and the total number of bases was 147,017,334,834. The GC content was 37.4% and the Q30 value was 90.45%. Trimmomatic was used to remove adapter sequences and low-quality reads to reduce biases in the analysis. After filtering the DNA sample, 749,527,438 reads were generated for a total of 112,735,647,909 bases. The GC content was 36.69% and the Q30 value was 96.49% (Table 1). If the amount of filtered data is more than 150-fold the genome size estimated by the genome survey, then the data were randomly subsampled to 150-fold the genome size in order to reduce the negative effect of too much data. The base quality plot generated by FastQC was used to check the overall quality of the produced data. This plot shows the range of quality values at each cycle. The x-axis and y-axis are the number of cycles and Phred quality score (Q-score), respectively (Figure 2).

### 3.3. K-Mer Analysis

**Figure 3:**
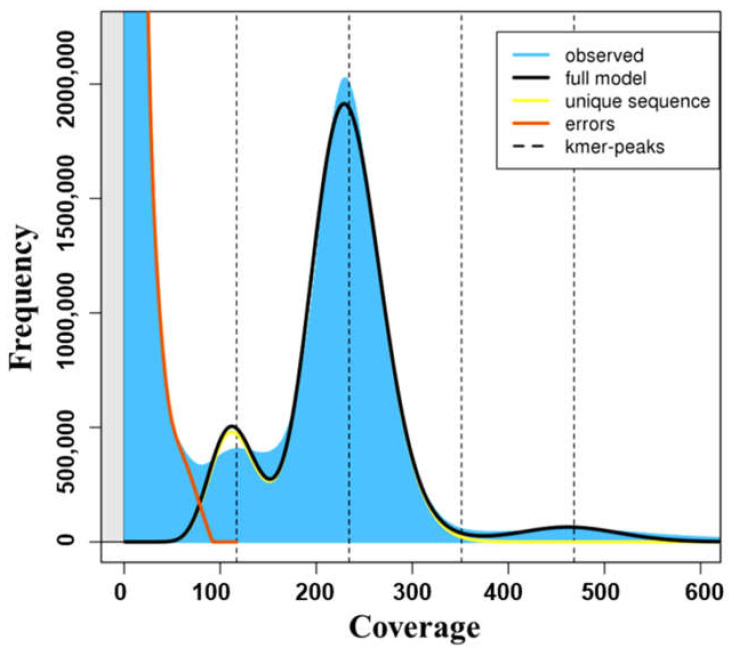
K-mer graph. The genome size can be estimated using the total k-mer number and volume peak. The top of the peak does not intersect the kmer-peak line because of overdispersion of the real data.

Prior to assembly, k-mer analysis was performed to estimate the genome size of the sample. Sharp left peaks indicate random sequencing errors, right peaks indicate adequate data and genome size can be estimated using total k-mer counts and volume peaks (Figure 3). The genome size estimation for Sample 3 using k-mer analysis is presented in Figure 3. A k-mer size of 21 was used for genome size estimation. A peak measured at half the peak depth showed a heterozygosity of 0.404 for Sample 3. The genomic sequence repeat length for Sample 3 was obtained by calculating the ratio of 1.8-fold the number of k-mers after the main peak to the total number of k-mers (Table 2).

### 3.4. Assembly Results

The best k-mer was selected by the status of the assembled results such as the number of contigs, contig sum and contig N50. As shown in Table 3, the number of identified contigs was 19,521, with a total of 298,515,080 bases. The contig N50 was 42,020. An N50 means that half of all bases reside in contigs of this size or longer. The longest contig was 411,840, the shortest was 1000 and the average length of the contigs was 15,260. Additionally, in the case of the assembled scaffolds, 14,625 scaffolds with 318,741,328 bases were identified, the N50 value was 78,430 and the average scaffold length was 21,794. The longest scaffold was 803,009 and the shortest scaffold was 1000.

### 3.5. Self-Mapping Results

To determine the insert size of the raw data and how many reads were used in the assembly, raw reads were mapped to the assembly results. As shown in Table 4, a total of 749,527,438 bases were read, and the number of bases read in the mapping was 655,896,731, accounting for approximately 87.51%. After mapping, the necessary statistics were calculated. The depth representing the number of times each gene was sequenced was calculated to be 276.51. When the depth is 80–150× of the genome size, it is possible to guarantee high accuracy and reliability by preventing substitution of the wrong nucleotide due to sequencing error; however, too great a depth (higher than 200×) can cause negative effects during assembly because sequencing error bases with high depth cannot be distinguished from normal bases. The percentage of the genomes covered by the reads was 94.65%. The adapter sequences were removed from the library, which consists of paired-end reads (Read 1, Read 2) and internal mate regions. The insert size of the sequence calculated from the mapping result was 475 bp. In general, inserts of paired-end libraries have sizes between 350 and 450 bp and are distributed in the form of a standard normal distribution.

### 3.6. Blast Results

**Figure 4:**
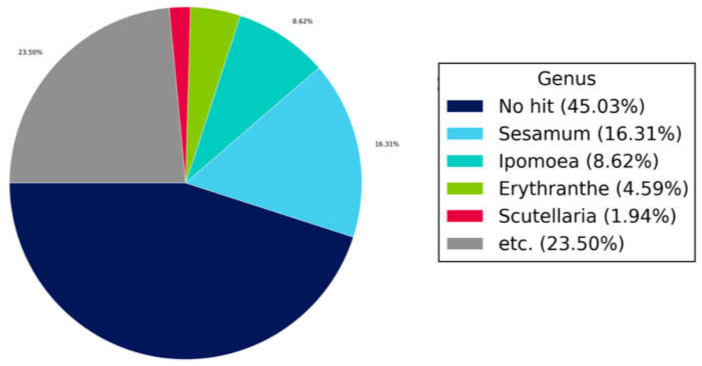
Genus-level summary of BLASTN results. The following figure shows the proportion based on the genus level as the result of the best hit for the entire contig.

After the complete genome or draft genome was assembled, BLAST analysis was carried out to identify to which species each scaffold showed similarity. The best hit and top five hit results were identified using the NCBI NT database. As a result of BLAST, 45.03% of the samples had no hits and the *Sesamum* genus was the most matched, with a rate of 16.31%. *Ipomoea* showed a rate of 8.62%, *Erythranthe* a rate of 4.59%, *Scutellaria* a rate of 1.94% and other genera, a rate of 23.50% (Figure 4).

### 3.7. BUSCO Results

To assess the completeness of the genome assembly, Benchmarking Universal Single-Copy Orthologs (BUSCO) analysis was performed based on evolutionarily informed expectations of gene content from near-universal single-copy orthologs. By default, bacteria or eukaryotic DB was used for analysis. As shown in Table 5, 303 total BUSCO groups were searched. There were 279 complete BUSCOs (92.08%). Of these, 229 single-copy BUSCOs (75.58%) and 50 duplicate-copy BUSCOs (16.50%) were analyzed. The number of fragmented BUSCOs, which were only partially recovered matches, was 4 (1.32%). There were 20 missing BUSCOs without a match, representing a rate of 6.60%.

### 3.8. Gene Prediction and Annotation Results

**Figure 5:**
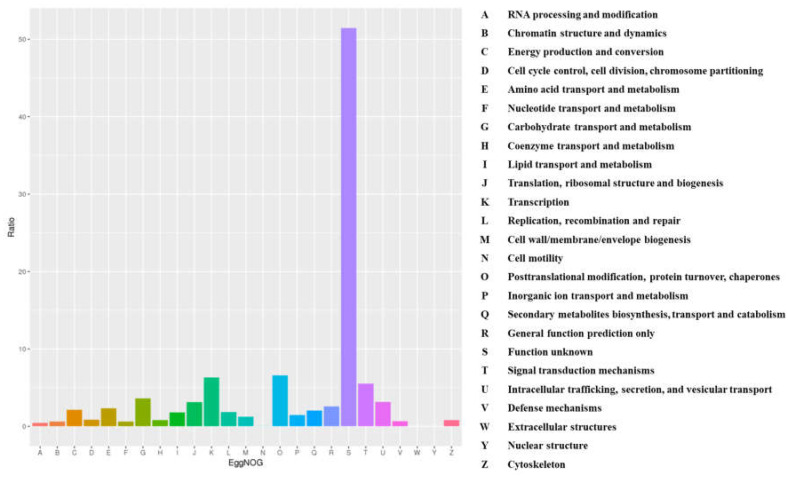
EggNOG functional classification of *Scutellaria indica* var. *tsusimensis* unigenes. In total, 23,716 unigenes were assigned to 25 categories.

After the complete genome or draft genome was assembled, the locations of protein-coding genes were predicted, and their functions were annotated. MAKER was used to predict genes in the draft genome, and the InterProScan, UniProt and EggNOG databases were used to annotate the functions of the genes. As shown in Figure 5, a total of 23,814 genes were identified by MAKER. The numbers of identified CDSs, tRNAs and rRNAs were 22,788, 670 and 356, respectively (Table 6). In EggNOG functional classification, a total of 23,716 unigenes were classified into 25 categories. Among them, “function unknown” was the largest group (12,200, 51.44%). This was followed by “Posttranslational modification, protein turnover, chaperones” (1565, 6.59%), “Transcription” (1500, 6.32%), “Signal transduction mechanisms” (1312, 5.53%) and “Carbohydrate transport and metabolism” (3861, 5.15%).

### 3.9. Validation of Species-Specific Primers Using Barcode DNA

**Figure 6:**
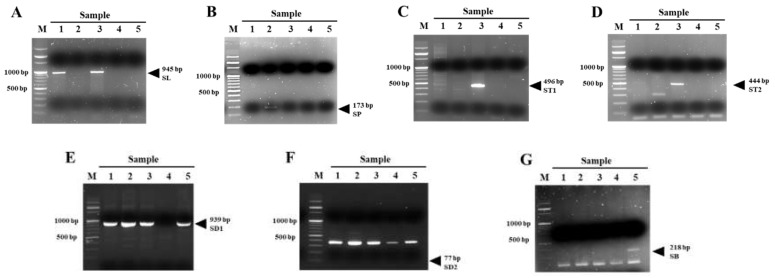
PCR analysis of five *Scutellaria* species using primers designed to amplify SL (**A**), SP (**B**), ST1 (**C**), ST2 (**D**), SD1 (**E**), SD2 (**F**) and SB (**G**). Amplicons: M, 100 bp DNA ladder; Sample 1, *Scutellaria indica* L.; Sample 2, *Scutellaria pekinensis* var. *transitra* (Makino) H. Hara; Sample 3, *Scutellaria indica* var. *tsusimensis* (H. Hara) Ohwi; Sample 4: *Scutellaria barbata* D. Don; Sample 5: *Scutellaria baicalensis* Georgi.

The assembled DNA was searched for species-specific primers. As a result of BLAST, nine chloroplast DNA loci were selected among 279 nucleotides and annotated genes matched in *Scutellaria* species. All the transcripts containing detected nucleotides and chloroplast DNA were subjected to primer design with Primer3. Among the resulting large number of primer pairs, 4 primers that specifically amplified DNA in 3 species were selected (Table 7). As shown in Figure 6A, the SL primer (945 bp) amplified DNA for both CYC2B of *S. indica* L. and *S. indica* var. *tsusimensis* (H. Hara) Ohwi. The SP primer (173 bp) amplified DNA specifically for PsbA of *S. pekinensis* var. *transitra* (Makino) H. Hara (Figure 6B). The ST1 and ST2 primers (491 bp and 444 bp, respectively) amplified DNA specifically for atpI_0 and rbcL_3, respectively, of *S. indica* var. *tsusimensis* (H. Hara) Ohwi (Figure 6C,D). The SD1 and SD2 primers (939 bp and 77 bp, respectively) did not amplify sequences specific to *S. barbata* D. Don (Figure 6E,F). The SB primers (218 bp) amplified chloroplast DNA of *S. baicalensis* Georgi (Figure 6G). Although no primers specifically amplified DNA in *S. indica* L. and *S. barbata* D. Don, the species could be distinguished by cross-validation with other primers. Differentiation methods were established for 5 species of the *Scutellaria* genus using the above primers. Table 7 provides the sequence and information of each primer. In conclusion, the leaves of each sample shown in Figure 1 could be characterized by specific primer sets and cross-validation. Sample 1 was *S. indica* L. and Sample 2 was determined to be *S. pekinensis* var. *transitra* (Makino) H. Hara. Sample 3 was judged to be *S. indica* var. *tsusimensis* (H. Hara) Ohwi and Sample 4 was judged to be *S. barbata* D. Don. Finally, Sample 5 was determined to be *S. baicalensis* Georgi.

## 4. Discussion

Recently, genetic investigations and the development of markers for subspecies of plants that cannot be classified visually or phenotypically have been active. Even for similar subspecies, the differences in physiological activity and components are clear, so these studies are receiving increasing attention [26,27]. In the field of marker development, a comprehensive investigation of new or combined species-specific variable markers in the chloroplast region of plants has begun. However, genetic information of plants is essential for differentiation and research is almost impossible if there is no reference genome. In this study, we aimed to genetically differentiate *S. indica* L. and *S. indica* var. *tsusimensis* (H. Hara) Ohwi, which are difficult to classify by phenotype. In some cases, most of the specimens identified as *S. indica* L. were actually *S. indica* var. *tsusimensis* (H. Hara) Ohwi [2]. In the NCBI GenBank database, about 17 species in the genus *Scutellaria* L. have reported the complete chloroplast genomes. However, among the five species of *Scutellaria* L. selected for this study, only *S. baicalensis* Georgi has been reported as a complete chloroplast genome. A specific primer developed using the reference genome was tested but classification failed because it did not show specific amplification. For this reason, we performed whole-genome de novo assembly to obtain the genome of the subspecies *S. indica* var. *tsusimensis* (H. Hara) Ohwi. Most of the genes did not have hits and 279 genes matched the genus *Scutellaria*. Primers were prepared by screening these 279 genes and nine annotated chloroplast barcoded DNA loci. Since the primers prepared with each sequence registered in GenBank did not show specific amplification, we aimed to design primers that were specific to each species by using the gene matched with the genus *Scutellaria* via BLAST. As a result, four specifically amplifying primers were designed and three species could be classified. The SP primer was specific to *S. pekinensis* var. *transitra* (Makino) H. Hara (*S. pekinensis* var. *transitra* (Makino) H. Hara voucher KUS:2006-1082 trnH-PsbA intergenic spacer, partial sequence; and PsbA (psbA) gene, partial CDS). It was prepared using the base sequence of the chloroplast (KX060016.1). Primers (ST1 and ST2 primers) specific to *S. indica* var. *tsusimensis* (H. Hara) Ohwi were prepared using the annotated chloroplast barcode DNA AtpI_0 and rbCL_3 nucleotide sequences. The sequences of these two barcode DNAs were registered in the NCBI GenBank database and given accession numbers MZ714561 and MZ714562, respectively. For *S. baicalensis* Georgi, the complete chloroplast genome sequence (*S. baicalensis* Georgi isolate BOP028266 chloroplast, complete genome (MF521633.1)) was deposited in GenBank, so it was possible to obtain the specific SB primer. The SL primer was produced using the nucleotide sequence of the *S. indica* L. TCP transcription factor (CYC2B) gene, partial CDS (KM526800.1), but amplified DNA in two species, *S. indica* L. and *S. indica* var. *tsusimensis* (H. Hara) Ohwi. However, when this primer was used with the ST1 or ST2 primer, *S. indica* L. could be discerned. The SD1 primer amplifies DNA in the *Scutellaria insignis* chloroplast, the complete genome (KT750009.1) was produced using the sequence and classification was possible because the primer amplified DNA only in *S. barbata* D. Don. However, it was concluded that this result alone was insufficient evidence, and a differentiation method was developed using both the SD2 and SD1 primers, which amplified DNA in all five species. The SD2 primer is specific to *Scutellaria indica* var. *tsusimensis* voucher SWUS (Kim 2008-008 psbK-psbI intergenic spacer, partial sequence). It was prepared using the chloroplast (KX059898.1) base sequence. This study focused on classifying five species of the genus *Scutellaria* L. native to Korea as a precedent study for our future research. Prior to this study, five species whose usefulness was confirmed in the screening process for new drug development were selected. For in-depth studies, it was necessary to investigate and standardize the active compounds for each of the five species. Except for *S. barbata* D. Don and *S. baicalensis* Georgi, the morphologies between the three species were very similar. Due to the similar shape of the three species, it was difficult to conduct research because it was difficult to supply and sample accurate raw materials. Therefore, research on classification and identification by species was urgently needed. Species-specific primer development was attempted by comparing each gene, but the three species lacked reference genomes and did not overlap, so SNP or barcode genes could not be targeted and failed to generate classification markers using the reported reference genome. Species-specific primers were prepared using the annotated cpDNA portion of the WGS results, *S. indica* L. and *S. indica* var. *tsusimensis* (H. Hara) Ohwi could be classified, and a classification method for five species was made with the additionally prepared primers. However, genetic studies on *S. indica* var. *tsusimensis* (H. Hara) Ohwi, *S. indica* L. and *S. pekinensis* var. *transtra* (Makino) H. Hara are still lacking, and further studies are needed. In addition, it cannot be regarded as a perfect genetic classification because species-specific primers focusing on the SNP part were not prepared by comparing cpDNA or barcode genes with each of the five overlapping species. Nevertheless, it is very meaningful to be able to classify five species of *Scutellaria* L. genus and we will make up for the shortcomings through future genetic research.

## 5. Conclusions

This study reports the sequencing, assembly and annotation of the genome of *S. indica* var. *tsusimensis* (H. Hara) Ohwi using de novo assembly, as well as a method for discriminating five species of *Scutellaria* using species-specific primers. Among the nucleotide sequences obtained as a result of WGS, annotated chloroplast DNA (AtpI_0, rbcL_3) was used to prepare a primer that was specific to *S. indica* var. *tsusimensis* (H. Hara) Ohwi, and using this primer, *S. indica* L. and *S. indica* var. *tsusimensis* (H. Hara) Ohwi were molecularly classified. In addition, a primer set that can distinguish among *S. pekinensis* var. *transitra* (Makino) H. Hara, *S. barbata* D. Don and *S. baicalensis* Georgi was developed. The results will lay a solid foundation for future genetic studies in this species. Furthermore, fractionation by species-specific markers identified in the assembled genome suggested that the markers will be useful for subsequent investigations of phylogenetic structure and genetic diversity.

## Figures and Tables

**Table 1:** Summary of the *Scutellaria indica* var. *tsusimensis* data.

	Raw Data Stats	Filtered Data Stats
Total read bases	147,017,334,834	112,735,647,909
Total reads	973,24,734	749,527,438
GC (%)	37.4	36.69
Q20 (%)	95.51	98.91
Q30 (%)	90.45	96.49

**Table 2:** K-mer analysis results.

	K-Mer Coverage	Heterozygosity	Genome Length	Genome Repeat Length
21mer	234.2	0.404	352,670,975	166,169,989

**Table 3:** Assembly summary of contigs and scaffolds.

	Contigs	Scaffolds
No.	19,561	14,625
Sum	298,515,080	318,741,328
N50	42,020	78,430
Longest	411,840	803,009
Shortest	1000	1000
Average length	15,260	21,794

**Table 4:** Overall mapping statistics.

Library Name	Total Reads	Mapped Reads	Coverage (%)	Depth	Ins. Size (Std.)
DNA	749,527,438	655,896,731 (87.51%)	94.65	276.51	475.88 (113.04)

**Table 5:** BUSCO analysis results.

Status	Of BUSCOs	Percentage
Complete BUSCOs (C)		
Complete and single-copy BUSCOs (S)	229	75.58%
Complete and duplicated BUSCOs (D)	50	16.50%
Fragmented BUSCOs (F)	4	1.32%
Missing BUSCOs (M)	20	6.60%
Total BUSCO groups searched	303	100.00%

**Table 6:** Genome annotation summary.

Sample	Contigs	Bases	Genes	CDSs	tRNAs	rRNAs
DNA	14,625	318,741,328	23,814	22,788	670	356

**Table 7:** Primer pairs tested by PCR with genomic DNA from five *Scutellaria* species.

Primer Name	Sequence (5′->3′)		Product Size	Tm (℃)	NCBI Accession of Target Genome	Locus	Target Species
SL Primer	Forward	TGCTTACCTGCTTCCACAGG	945 bp	54	KM526800.1	*CYC2B*	*Scutellaria indica* L.
	Reverse	TCGGTGGCGACGTTATATGG					
SP Primer	Forward	GAAATTACTTTTAAATTCAT	173 bp	42	KX060016.1	*trnH-PsbA*	*Scutellaria pekinensis* var. *transitra* (Makino) H. Hara
	Reverse	GTAGTCTTTCCTAGACTTTA					
ST1 Primer	Forward	TTGTGGCATCACTAACCCCC	491 bp	54	MZ714561	*AtpI_*0	*Scutellaria indica* var. *tsusimensis* (H. Hara) Ohwi
	Reverse	AGGGGTAGGCTGAACGTACT					
ST2 Primer	Forward	CGCATTCCTCCAGCCTATGT	444 bp	54	MZ714562	*rbcL_*3	*Scutellaria indica* var. *tsusimensis* (H. Hara) Ohwi
	Reverse	ATCACGGCAGTAGTGTGCAA					
SD1 Primer	Forward	ATAACTTCCCTCTAGACTTA	939 bp	43	KT750009.1	-	*Scutellaria barbata* D. Don
	Reverse	TGAATTTCAATTATTTTTTC					
SD2 Primer	Forward	ACCCTTGATTCGCACACTGA	77 bp	55	KX059898.1	*PsbK-PsbI*	*Scutellaria barbata* D. Don
	Reverse	TGAGGAAACGGACGTAAGCC					
SB Primer	Forward	TCCCCAAAAAGTGGATCCCG	218 bp	55	MF521633.1	-	*Scutellaria baicalensis* Georgi
	Reverse	GGGCCTCATTGGTAAGTGCT					

## Data Availability

Data are available from the corresponding author upon specific request.
